# Zebra Fish Lacking Adaptive Immunity Acquire an Antiviral Alert State Characterized by Upregulated Gene Expression of Apoptosis, Multigene Families, and Interferon-Related Genes

**DOI:** 10.3389/fimmu.2017.00121

**Published:** 2017-02-13

**Authors:** Pablo García-Valtanen, Alicia Martínez-López, Azucena López-Muñoz, Melissa Bello-Perez, Regla M. Medina-Gali, María del Mar Ortega-Villaizán, Monica Varela, Antonio Figueras, Víctoriano Mulero, Beatriz Novoa, Amparo Estepa, Julio Coll

**Affiliations:** ^1^Departamento de Bioquímica, Universidad Miguel Hernández de Elche (UMH), Alicante, Spain; ^2^Facultad de Biología, Departamento de Biología Celular e Histología, Universidad de Murcia, IMIB-Arrixaca, Murcia, Spain; ^3^Instituto de Investigaciones Marinas (IIM), Consejo Superior de Investigaciones Científicas (CSIC), Vigo, Spain; ^4^Departamento de Biotecnología, Instituto Nacional Investigación y Tecnología Agraria y Alimentaria (INIA), Madrid, Spain

**Keywords:** zebra fish *rag1*^−/−^ adaptive deficient mutants, spring viremia carp viral infections, multigene families and apoptosis in resistance to viral infections, trained immunity NK/macrophages in fish, antiviral alert state

## Abstract

To investigate fish innate immunity, we have conducted organ and cell immune-related transcriptomic as well as immunohistologic analysis in mutant zebra fish (*Danio rerio*) lacking adaptive immunity (*rag1*^−/−^) at different developmental stages (egg, larvae, and adult), before and after infection with spring viremia carp virus (SVCV). The results revealed that, compared to immunocompetent zebra fish (*rag1^+/+^*), *rag1*^−/−^ acquired increased resistance to SVCV with age, correlating with elevated transcript levels of immune genes in skin/fins and lymphoid organs (head kidney and spleen). Gene sets corresponding to apoptotic functions, immune-related multigene families, and interferon-related genes were constitutively upregulated in uninfected adult *rag1*^−/−^ zebra fish. Overexpression of activated CASPASE-3 in different tissues before and after infection with SVCV further confirmed increased apoptotic function in *rag1*^−/−^ zebra fish. Concurrently, staining of different tissue samples with a pan-leukocyte antibody marker showed abundant leukocyte infiltrations in SVCV-infected *rag1*^−/−^ fish, coinciding with increased transcript expression of genes related to NK-cells and macrophages, suggesting that these genes played a key role in the enhanced immune response of *rag1*^−/−^ zebra fish to SVCV lethal infection. Overall, we present evidence that indicates that *rag1*^−/−^ zebra fish acquire an antiviral alert state while they reach adulthood in the absence of adaptive immunity. This antiviral state was characterized by (i) a more rapid response to viral infection, which resulted in increased survival, (ii) the involvement of NK-cell- and macrophage-mediated transcript responses rather than B- and/or T-cell dependent cells, and (iii) enhanced apoptosis, described here for the first time, as well as the similar modulation of multigene family/interferon-related genes previously associated to fish that survived lethal viral infections. From this and other studies, it might be concluded that some of the characteristics of mammalian trained immunity are present in lower vertebrates.

## Introduction

Immunity against infections in vertebrate species includes both innate (early and unspecific) and adaptive (late and specific) responses. However, how these responses interact to exert coordinated immune responses during infection remains poorly understood, especially in primitive vertebrates such as fish. In contrast to the mammalian immune system, fish have only tetrameric IgM in their sera, which does not undergo IgM affinity maturation or isotype switch, elicit rapid but less-efficient adaptive secondary responses (1), and possess mucosal IgT and unique phagocytic B-cells (2). On the other hand, protection against fish pathogens seems to rely more heavily on innate rather than adaptive responses (2–4). Thus, fish species are suitable models to study the specific role of innate immunity during infections.

Recent research conducted in mammal species has revealed that cells of the innate immune system can be primed so that upon a secondary immune challenge they are capable of eliciting more efficient immune responses, a characteristic previously attributed only to the adaptive arm of the immune system. These types of innate responses have been termed trained immunity (5–10). Mammalian trained immunity has the following properties: (a) it enhances the speed and magnitude of the responses to second pathogen encounter similar to adaptive immunity, (b) it is acquired after exposure to a pathogen, not inherited, (c) it protects against unrelated pathogens, (d) it is mediated by macrophages (11, 12) and/or natural killer (NK)-cells (13–16) rather than by lymphocytes (17–19), and (e) it is generated by epigenetic reprogramming (alternative splicing, DNA/histone modifications, miRNA, etc.) rather than by genetic recombination (12, 20). Trained immunity phenomena are largely unexplored in fish species despite the fact that these primitive vertebrates offer suitable models to study the innate immune system. Nevertheless, some examples of innate immune responses presenting characteristics similar to those of mammalian trained immunity have been reported. For instance, salmonid vaccines against novirhabdoviruses also protect against unrelated nodaviruses (21) or spring viremia carp virus (SVCV) (22). Likewise, β-glucans, widely present in the cell wall of bacteria and fungi, have long-term effects on fish innate immune responses (23). Additionally, mutant zebra fish lacking adaptive immune responses maintain protective immune memory against secondary bacterial infections (24, 25), and the modulation of several innate immune multigene families was implicated in the rapid memory responses to rhabdoviral infection in fish that had previously survived infection with the same virus (26, 27). Further characterization of trained immunity or its equivalent innate immune response in fish species has the potential to drive research to develop new vaccine concepts. Thus, traditionally, successful vaccines candidates for vertebrate species are selected on the basis that they both contain the pathogen antigen(s) that elicit strong pathogen-specific T and B cell-mediated responses and confer protection. With increasing evidence that innate, unspecific protective responses are also elicited by some vaccines (28, 29), the possibility to develop vaccines against wider pathogen spectra (non-specific or heterologous vaccines) is actively being explored (30–32). For instance, non-specific vaccines could be obtained by (i) triggering still unknown multiple receptors to improve the simultaneous recognition of different pathogens (32) and/or generate immune synergies (33, 34), (ii) activating long-term specific NK cells to increase IFNγ and Th1 responses (13–15, 35), and/or (iii) designing novel molecular adjuvants. Innate trained immunity is likely to mediate some of vaccine unspecific, protective effects associated to epigenetic changes (19, 29, 36). Such non-specific vaccine strategies will be most practical for farmed fish vaccination because with short lifespans, heterologous vaccines would represent a more cost-effective alternative than traditional vaccines.

Mutant *rag1*^−/−^ zebra fish (37) offer a new opportunity to explore innate responses to pathogens in the absence of adaptive immunity (24) and therefore explore trained immunity and new vaccination strategies. These mutants were generated by introducing a premature stop codon at *rag1t26683*, which resulted in the expression of a truncated, inactivated form of RAG1 (38, 39). Zebra fish *rag1*^−/−^ mutants fail to undergo germ line V(D)J recombination in both immunoglobulin (*ig*) and T-cell receptor (*tcr*) gene variable segments (40–42). In turn, this leads to the absence of mature *igs/tcr* transcripts and reduced B- and T-cell numbers in lymphoid organs (head kidney and spleen), rendering *rag1*^−/−^ zebra fish deficient in adaptive immunity (43–45). Despite this and the problems associated with breeding these fish compared with similar mutants in mice (46), *rag1*^−/−^ zebra fish are capable of surviving in non-sterile aquarium facilities (47). In addition to this, *rag1*^−/−^ zebra fish exhibit relatively enhanced immune responses to bacterial infections (25, 48, 49). However, whether the overall immune phenotype resulting from the *rag1*^−/−^ genotype is acquired or inherited and whether similar phenotypes are also acquired after fish viral infections remains unclear. To investigate this, we have used *rag1*^−/−^ zebra fish to correlate resistance to viral infection with innate gene expression levels (transcriptomic and cellular responses) in eggs, larvae, and adult *rag1*^−/−^ zebra fish. Probably, due to the difficulties encountered when breeding *rag1*^−/−^ zebra fish, their gene expression profiles, including changes in their transcriptome in response to viral infection, has yet to be compared to *rag1^+/+^* (49). Here, we have successfully raised enough numbers of *rag1*^−/−^ zebra fish and compared their gene expression profiles in response to SVCV with those of *rag1^+/+^*.

For this work, SVCV was chosen for the infection model because zebra fish are susceptible to this pathogen (50, 51), and the transcriptomic responses of zebra fish to SVCV infection by bath immersion have been investigated (52). SVCV is a rhabdovirus, recently classified within the *Sprivivirus* genus (53) that naturally infects cyprinid species, mainly carp species such as *Cyprinus carpio* (54, 55). The progress of the SVCV infection is externally associated with exophthalmia, abdominal distension, and petechial hemorrhages of the skin and gills. Moreover, most important fish lymphoid organs such as head kidney, spleen, and liver are also affected (55, 56). As with other rhabdoviruses, SVCV enters the fish *via* the base of the fins and skin (57). These tissues are of special importance in the initial response to infection of *rag1*^−/−^ by mycobacterial (47) or SVCV (17, 51). High zebra fish mortalities occur 5–10 days after SVCV water-born infection at 10–17°C, but at 26°C infected fish generate neutralizing antibodies increasing survival to the disease (52). At later stages of infection, SVCV virions are shed mostly with the feces and urine and may infect other fish (55, 56). *In vitro* replication of SVCV takes place in the cytoplasm of cells from different origins, including mammalian cells, but to obtain replication in these cells, temperatures must be maintained within 10–30°C with optimal virus growth at 20°C (56).

Our results suggest that while aging, and particularly during the period of time when *rag1*^−/−^ zebra fish reach adulthood, the innate immune system gradually shifts from one that is underdeveloped and incapable of eliciting protective responses against SVCV infection (in the egg and larval stages) to one that exerts enhanced, antiviral protective responses, compared to adult age-matched *rag1^+/+^* zebra fish. This acquired antiviral alert state was characterized by constitutively upregulated transcripts (i.e., *fas, fasl, hsp90, casp7*, and *hspb*) and protein (activated CASPASE-3) levels of apoptosis effector molecules, which is described here for the first time. Additionally, *rag1*^−/−^ zebra fish presented abundant infiltration of leukocytes in different non-lymphoid organs, and this coincided with elevated transcript expression of NK-cell, macrophage, apoptosis-, immune-related multigene families, and interferon-related genes. Partial resistance to SVCV challenge observed in naïve adult *rag1*^−/−^ zebra fish suggests that in these fish innate immunity was enhanced and was able to mount efficient responses shortly after exposure to SVCV by bath immersion, contrary to their immunocompetent counterparts. Acquired, non-specific, enhanced innate immune responses in *rag1*^−/−^ zebra fish resemble trained immunity responses in other vertebrate species. While the mechanisms of trained immunity or its equivalent in fish species have not been described yet, our work shows that the occurrence of similar immune phenomena is facilitated in *rag1*^−/−^ zebra fish, indicating that this model could be particularly well suited for further studies of these immunological responses.

## Materials and Methods

### Zebra Fish (*Danio rerio*)

Wild-type AB family founder zebra fish (*D. rerio*) were originally obtained from David Raible’s fish facility at the University of Washington (USA). Mutant adult zebra fish recombinant activation gene (*rag1*^−/−^) and wild type (*rag1^+/+^*) were reproduced and characterized at the University of Murcia (Dr. Víctoriano Mulero). Adult zebra fish *rag1*^−/−^ and *rag1^+/+^* were raised and genotyped when they reached 0.5–1 g (~6 months of age). Figure S1 in Supplementary Material shows the smaller size and apparent accelerated aging in *rag1*^−/−^ fish, compared to *rag1^+/+^* fish. As we have experienced in three different laboratories (CSIC, UM, and UMH), *rag1*^−/−^ zebra fish mutants are difficult to raise and even more to reproduce compared to similar mutants in mice (46). These difficulties may explain why few people could make experiments with them and why only heterozygous *rag1^±^* rather than homozygous *rag1*^−/−^ have been used for microarray analysis (49). Zebra fish were maintained at 28°C in 30 l aquaria with tap-dechlorinated carbon-filtered water with 1 g of CaCl_2_, 1 g of NaHCO_3_, and 0.5 g of Instant Ocean sea salts added to water resulting in a conductivity of 200–300 μS and pH of 7.8–8.2. The aquaria were provided with biological filters and fish fed daily with a commercial feed diet. Previously to the infection experiments, all fish were acclimatized to 22°C for 2 weeks.

### ZF4 Cell Culture and SVCV

The zebra fish embryonic fibroblast (ZF4) cell line (58) was purchased from the American Type Culture Collection (number CRL-2050). ZF4 cells were maintained at 28°C in a 5% CO_2_ atmosphere in RPMI 1640-Dutch modified culture medium (Gibco, Invitrogen Co., UK) supplemented with 20 mM HEPES, 10% fetal calf serum (Sigma, St. Louis, MO, USA), 1 mM piruvate, 2 mM glutamine, 50 μg/ml of gentamicin, and 2.5 μg/ml of fungizone.

The SVCV isolate 56/70 (59, 60) was grown in ZF4 cells at 22°C in the presence of 2% fetal calf serum. Supernatants from ZF4 cell monolayers infected with SVCV were clarified by centrifugation at 4,000 × *g* for 30 min and kept in aliquots at −70°C until used as described before (22, 61, 62). Viral titers of SVCV were determined by methylcellulose plaque assays (56). Briefly, ZF4 cell monolayers were infected with different dilutions of SVCV in 24-well plates for 90 min. Then, the cell culture media were removed, wells covered with 2% methyl cellulose (Sigma, St. Louis, USA) in cell culture media and plates incubated at 22°C. After 5 days, the media were removed and cell monolayers stained with 1% crystal violet-formalin to count plaque forming units (pfu). Please note that SVCV was recently renamed *Carp Sprivivirus* (53). However, to avoid confusion we have kept the traditional name for this publication.

### *In Vivo* Infection (Challenge) of Zebra Fish with SVCV

Spring viremia carp virus infections were conducted as in previous studies (22, 61, 62). Briefly, zebra fish were exposed to SVCV (10^3^, 10^4^, or 10^5^ pfu/ ml) by bath immersion for 90 min at 22°C (optimal temperature for SVCV replication). Mock-infected zebra fish were incubated with cell culture medium in parallel experiments. After SVCV infection, zebra fish were transferred to tanks with clean water and kept at 22°C to allow the progress of SVCV infection to until tissues were harvested or challenges ended.

Transcript expression folds were evaluated at 2 days after infection. At this time point, higher percentages of genes are differentially transcribed in virally infected fish (63–71), no new viruses are yet released into the water and external SVCV infection symptoms start to appear (52).

To evaluate mortalities, SVCV infections were allowed to proceed during 33 days. From days 2 to 33, infected and non-infected zebra fish were monitored daily to remove those fish that presented external hemorrhages.

### Ethic Statement of Zebra Fish Handling

During SVCV-induced mortalities, zebra fish were monitored 2–4 times per day and those with external hemorrhages killed by an overdose of anesthetics (methanesulfonate 3-aminobenzoic acid ethyl ester, MS-222) (Sigma-Aldrich) to minimize their suffering (72). Zebra fish were killed by an overdose of MS-222 to extract their tissues. To date, there is no evidence that short exposure to MS-222 has measurable effects on gene expression (since maximum changes are found >2 days of infection) and the use of differential expression fold calculations should eliminate any small differences; however, this cannot be completely rule out since it may affect different genes differentially. Fish were handled in accordance with the National and European guidelines and regulations on laboratory animal care. All the experiments were performed using protocols approved by the European Union Council Guidelines (86/609/EU). Animal work was approved by the UMH, CSIC, UMU, or INIA corresponding Ethic Committees.

### Determination of SVCV Titers in Zebra Fish Organs after Infection with SVCV

Viral titers in zebra fish were determined as described previously (61). Briefly, pooled internal organs, skin, and fins from four fish culled by exposure to MS-222 (see above) were disrupted and homogenized using a pestle, homogenized using a sterile nylon cell strainer (BD Falcon, MA, USA), resuspended in 3 ml of cell culture medium and passed through 0.2 μm sterile filters to remove bacterial contamination. Virus containing suspensions were assessed using the same methylcellulose method as above.

### RNA Isolation from Different Zebra Fish Tissues

To evaluated transcript expression in zebra fish organs by reverse transcriptase and quantitative polymerase chain reaction (RTqPCR), caudal and pectoral fins and adjacent skin were excised from four to six adult zebra fish per group before and after SVCV infection. Additionally, whole larvae and embryo eggs were pooled (*n* = 10–20 fish) in each group. RNA was extracted using the E.Z.N.A HP Tissue RNA kit (Omega Bio-tek, Norcross, GA, USA) following manufacturer’s instructions. Isolated RNAs were stored at −80°C until used.

For microarray analysis of *rag*^−/−^ and *rag^+/+^* zebra fish 2 (SVCV infection) or 4 [poly(I:C) injection] experiments were carried out. In each group, head kidney and spleen from individual zebra fish were pooled to obtain enough RNA for hybridization. Pooled head kidneys and spleens (*n* = 3 fish) were immediately immersed in RNAlater (Ambion, Austin, TX, USA) at 4°C overnight before being frozen at −70°C until processed. RNA was extracted from sonicated (1 min × 3 times at 40 W in ice) organs using a commercial RNA isolation kit (RNeasy kit, Qiagen, Hilden, Germany). RNA concentrations were estimated with Nanodrop and the presence of 18 and 28 S bands confirmed by denatured RNA agar electrophoresis (Sigma, Che. Co, MS, USA).

### Differential Expression of Selected Gene Set (sGS) by RTqPCR

To study innate/adaptive immune responses by RTqPCR in embryo eggs, larvae, and adult zebra fish in *rag1*^−/−^ versus *rag1^+/+^* groups, a selected gene set (sGS) was chosen as representative genes of innate and adaptive responses based in our own and also other studies. The sGS contained the following groups of immune-related genes: (i) pro-inflammatory cytokines interleukin-1β (*il1b*) and tumor necrosis α (*tnfa*), (ii) innate immunity-related genes such as the virus-induced transcription factor interferon regulatory factor-3 (*irf3*), tank-binding kinase-1 (*tbk1*), which induces IRF3 in mammalian models, tripartite-motif-21 (*trim21*), which directs virions to the proteasome for proteolysis, interferon PHI3 (*ifnphi3*) implicated in antiviral alert innate/adaptive responses in zebra fish, and interferon-induced myxovirus resistance isoforms A–B (*mxab*) and C (*mxc*) important in the zebra fish response to rhabdoviral infections (26), (iii) effector proteins such as the antimicrobial peptide defensin 2-like (*defbl2*), which exhibits antiviral properties against SVCV (73), and the antibacterial protein released from NK-cells NK-LYSIN (*nklysin*) and, (iv) adaptive immunity-related genes such as the markers of helper and cytotoxic T lymphocytes cluster of differentiation 4 and 8 (*cd4* and *cd8a*, respectively), NK cell- and T cell-generated interferon gamma (*ifng*) produced after viral infections and finally immunoglobulin M (*igm*) a marker of mature B-cells. Their corresponding primers are listed in Table S1 in Supplementary Material.

To perform RTqPCR assays 1 μg of RNA was used to obtain cDNA using reverse transcriptase (Moloney murine leukemia virus, Invitrogen) as previously described (73). Quantitative PCR was performed using ABI PRISM 7300 (Applied Biosystems, NJ, USA) and SYBR Green PCR master mix (Life Technologies, UK). Reactions were prepared in 20 μl volume with 2 μl of cDNA, 900 nM of each primer (Table S1 in Supplementary Material) and 10 μl of SYBR Green PCR master mix. Non-template controls were included for each gene analysis. The cycling conditions were 95°C for 10 min, followed by 40 cycles at 65°C 1 min, 95°C for 1 min, and extension of 10 min. The results were analyzed using the 2^−δδCt^ method (74). Each gene expression value was normalized by the formula, expression of each gene/expression of *ef1a*. Similar results were obtained using the sum of all gene expression values for normalization (not shown). Differential folds were then calculated by the formula, normalized expression of each *rag1*^−/−^ gene/normalized expression of each *rag1^+/+^* gene.

### Microarray Hybridization, GSEA, and mMPG Analysis

To target a larger amount of immune-related genes even those which are found in the lowest concentrations, we employed two formats of zebra fish 60-mer oligo microarrays: (i) immune-targeted in-house-designed microarray, Agilent’s ID47562 in a 8 × 15 K format-containing 14,541 fully annotated sequences, previously described, validated (26, 52), and deposited in Gene Expression Omnibus GEO’s GPL17670 (SVCV-infected zebra fish) and (ii) genome-wide commercial microarray Agilent’s ID019161 in a 4 × 44 K format vs2-containing 43,803 partially annotated sequences.

To account for the biological effects arising from small changes in several related genes, we used our previously designed 104 gene set (GS) collection (26). The GS collection was designed from immune-related sequences obtained from key-word searches at the GeneBank, KEGG, and WIKI human/zebra fish pathways (as accessed in 2012) plus new fish GS resulting from leading edge gene analysis (LEGA) of previously studied microarray results in VHSV (a rhabdovirus related to SVCV) survivors (26). Our GS collection was used here for analysis of *rag1*^−/−^ and *rag1^+/+^* by gene set enrichment analysis (GSEA) (http://www.broad.mit.edu/GSEA) (75–77). GSEA assigns a normalized enrichment score (NES) to each GS calculating its corresponding false discovery rates (FDR) for significance evaluation of differential expression folds. The most stringent cut-off value of <0.05 (**) or <0.25 FDR (*) were used in this work for NES to determine statistical significance.

Samples labeled from 2 μg of high quality RNA (50 μg/ml) were hybridized to the microarrays by Nimgenetics (Cantoblanco, Madrid, Spain). Raw and normalized data were deposited in the GEO bank at http://www.ncbi.nlm.nih.gov/geo/query/acc.cgi?acc, at GSE54096 (SVCV-infected zebra fish) for the experiments performed at the INIA laboratory and at GSE91397 [poly(I:C)-injected zebra fish] for the experiments performed at the CSIC laboratory. Biological replicates were obtained from head kidney/spleens from 3 pooled zebra fish per replica. Normalized data from two (SVCV-infection) or four [poly(I:C) injection] biological replicas were downsized to about 2,000 unique annotated genes after removing repeated and non-annotated genes and/or genes having outlier fold values. The resulting gene lists were compared and ranked by the *t*-test statistic metric to calculate GSEA NES. The different genotype or phenotypes comparisons were labeled as A, *rag1*^−/−^ versus *rag1^+/+^* genotypes; B, SVCV-infected or poly(I:C) injected *rag1*^−/−^ phenotypes versus *rag1*^−/−^ genotypes; or C, SVCV-infected or poly(I:C)-injected *rag1^+/+^* phenotypes versus *rag1^+/+^* genotype.

To search for modulated MultiPath Genes (mMPG), those genes present in >6 pathways were extracted as described before using the software Origin pro vs8.6 (Northampton, MA, USA) (52). After normalization, fluorescence outliers defined as values outside the means ± SDs, were first masked from mean calculations (*n* = 2). Folds were then calculated by applying the formula, normalized gene fluorescence value for each biological replica/non-infected mean. Fold outliers were then eliminated and their mean and SDs recalculated. Those MPG with folds >1.5 or <0.66, significant at the *p* > 0.05 level (*n* = 2 or 4) using the two-tail independent *t*-test, were considered modulated (mMPG).

### Cellular Gene Sets Defining Specific Immune-Related Cells

We used the cellular gene sets (cGS) described before (26), for Th1, T helper 1 cells; Th2, T helper 2 cells; Th17, T helper 17 cells; Treg, T regulatory cells; BZ cells, mucosal IgZ producing cells; B-cells, IgM-producing cells; Dendritic, dendritic cells; CTL, antigen-specific cytotoxic cells; NK-cells, NK cells; macrophages, monocyte and macrophages; and Neutrophil, neutrophil and granulocyte cells. To define the cGS, activating, membrane, and secreted genes were searched for each cellular type from different sources. The resulting cGS were used as inputs for GSEA.

### Histology and Immunohistochemistry

Zebra fish were euthanized with MS-222 (as described above) and a small incision made on the ventral body from the anus to the anterior part of the body cavity. They were then fixed in 10% buffered formalin, embedded in paraffin and cut in sections of 3 μm. Sections were stained with anti-human/mouse active CASPASE-3 antibody (R&D Systems, MN, USA), which recognizes a fully conserved epitope of zebra fish CASPASE-3 (78) or anti-human l-plastin antibody (Gene Tex, CA, USA). All sections were slightly counterstained with hematoxylin.

## Results

### Embryos and Larvae *rag1*^−/−^ Zebra Fish Do Not Possess Enhanced Innate Immunity

In non-sterile aquaria both *rag1*^−/−^ and *rag1^+/+^* zebra fish survive early stages of development (egg/larvae) and reach adulthood. However, adult fish in each group present differences in their physical appearance. Commonly, *rag1*^−/−^ are smaller and present features that are indicative of accelerated physical deterioration or aging (Figure S1 in Supplementary Material), in contrast to mice *rag1*^−/−^ mutants. The contrast between the cleanliness of mouse rooms and that of fish tanks may explain such difficulties. Based on the above mentioned observations, we asked the question, is the innate immune function at early stages of development different in *rag1*^−/−^ zebra fish?

To answer this question, we selected some immune genes that, based on our own and other publications, are regulated when zebra fish are exposed to pathogens. Our resulting selected gene set (sGS) included the genes of inflammatory cytokines (*il1b, tnfa*), innate immune transcription factors and effector molecules (*irf3, tbk1, trim21, ifnphi3, mxab, mxc*), antimicrobial peptides (*defbl2, nklysin*), and molecules involved in adaptive immune responses (*cd4, cd8, ifng, igm*).

**Figure 1:**
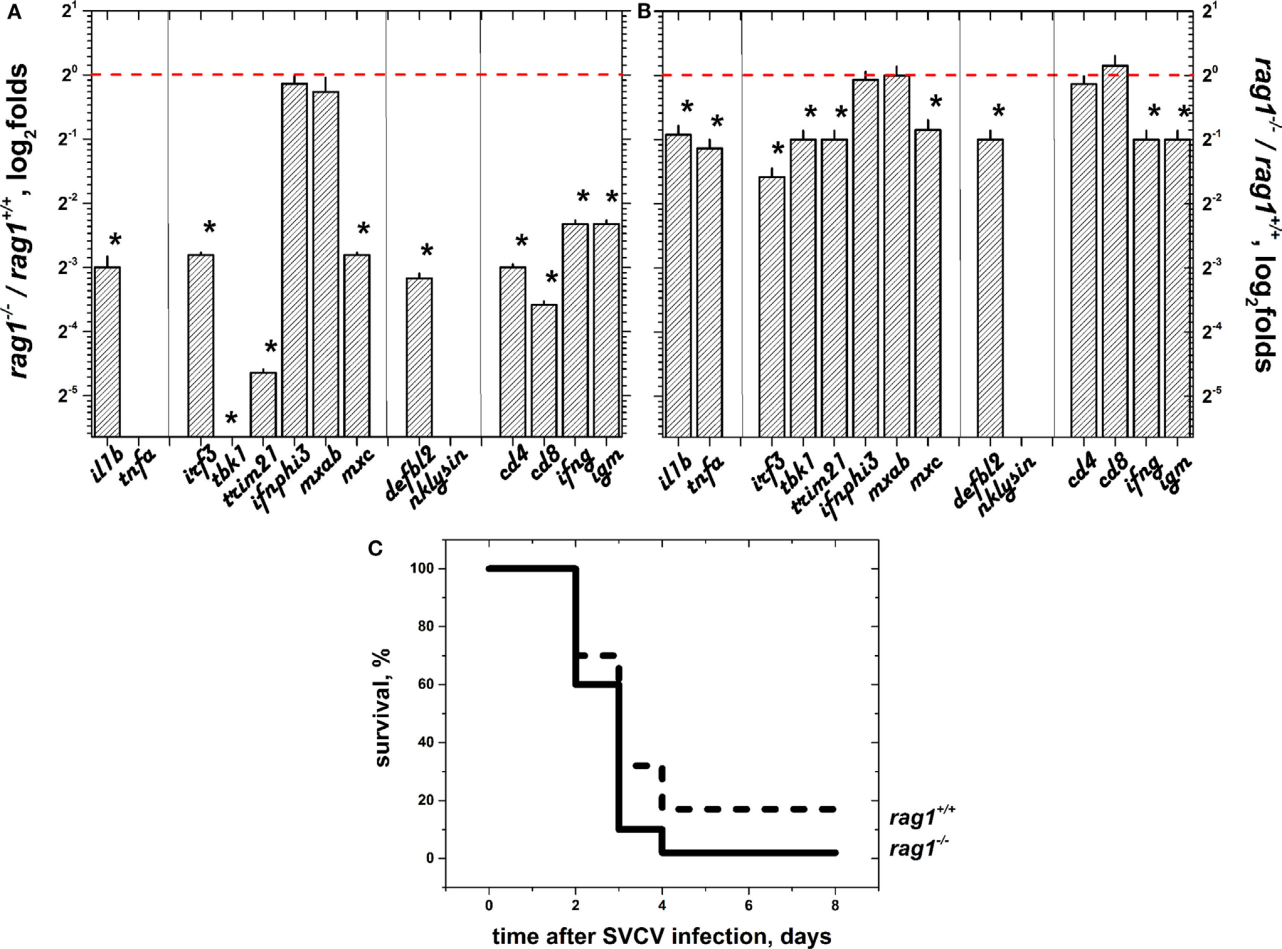
**Selected gene sets (sGS) were downregulated in *rag1*^−/−^ egg embryos but were similar in early hatched larvae and both showed high mortalities when infected by spring viremia carp virus (SVCV)**. **(A)** Expression folds in embryo eggs 1 day after fertilization (*n* = 60). Reverse transcriptase and quantitative polymerase chain reaction data were first normalized by the formula, expression of each gene/expression of *ef1a*. Differential folds were then calculated by the formula, normalized expression of each *rag1*^−/−^ gene/normalized expression of each *rag1^+/+^* gene*. Tbk1* fold was lower than 2^−6^ (not shown). *Tnfa* and *nklysin* were not done. Red dashed line, onefold boundary. *Statistically different from onefold with *p* < 0.05 by the *t*-test. **(B)** Expression folds in hatched larvae 3 days after fertilization (*n* = 45) calculated as in **(A)**. **(C)** Kaplan–Meier survival curves of naïve *rag1*^−/−^ (*n* = 90) and *rag*^+/+^ (*n* = 90) recently hatched larvae after bath infection in 10^4^ pfu of SVCV per ml (*n* = 2) at 22°C (optimal replication for SVCV). Differences between *rag1*^−/−^ and *rag^+^*^/+^ survival curves of SVCV-infected larvae as determined by the Gehan–Breslow–Wilcoxon test were significant with *p* < 0.05 (*). Solid lines, *rag1*^−/−^ larvae. Dashed lines, *rag^+^*^/+^ larvae.

Except for *ifnphi3*, and *mxab*, the all of the genes were downregulated (folds < 1) in *rag1*^−/−^ egg embryos at 24 h post-fertilization (hpf), compared to *rag1^+/+^* eggs (Figure 1A). This indicated that immune factors directly inherited from the mother were less efficiently passed or absent in *rag1*^−/−^ egg embryos, compared to embryos in the *rag1^+/+^*group. Similarly, we evaluated the transcript expression folds (*rag1*^−/−^ versus *rag1^+/+^*) in hatched larvae at 72 hpf. The differential expression in *rag1*^−/−^ larvae had increased relative to egg embryos, reaching levels similar to those of *rag1^+/+^* larvae (compare results from Figure 1A to those of Figure 1B). Next, we tested the resistance of zebra fish larvae to SVCV infection. In the conditions used for infection (10^4^ pfu of SVCV per ml, 22°C), both *rag1*^−/−^ and *rag1^+/+^* larvae were susceptible. However, survival rates were modest, 4.4% in *rag1*^−/−^ and 16.6% in *rag1^+/+^* (Figure 1C), similar to those previously reported for *rag1^+/+^* larvae (51).

The fact that neither egg embryos nor larvae in the *rag1*^−/−^ group showed upregulation in the expression of immune-related genes prior to SVCV infection or increased survival after SVCV infection, supports the idea that zebra fish need more time to develop an innate immune system to survive prolonged exposure to microorganisms in the water. To test this hypothesis, we then studied the innate immune response to SVCV infection in adult zebra fish.

### SVCV Infection of Adult Zebra Fish Shows That Naïve *rag1*^−/−^ Fish Are More Resistant Than Their *rag1^+/+^* Counterparts

**Figure 2:**
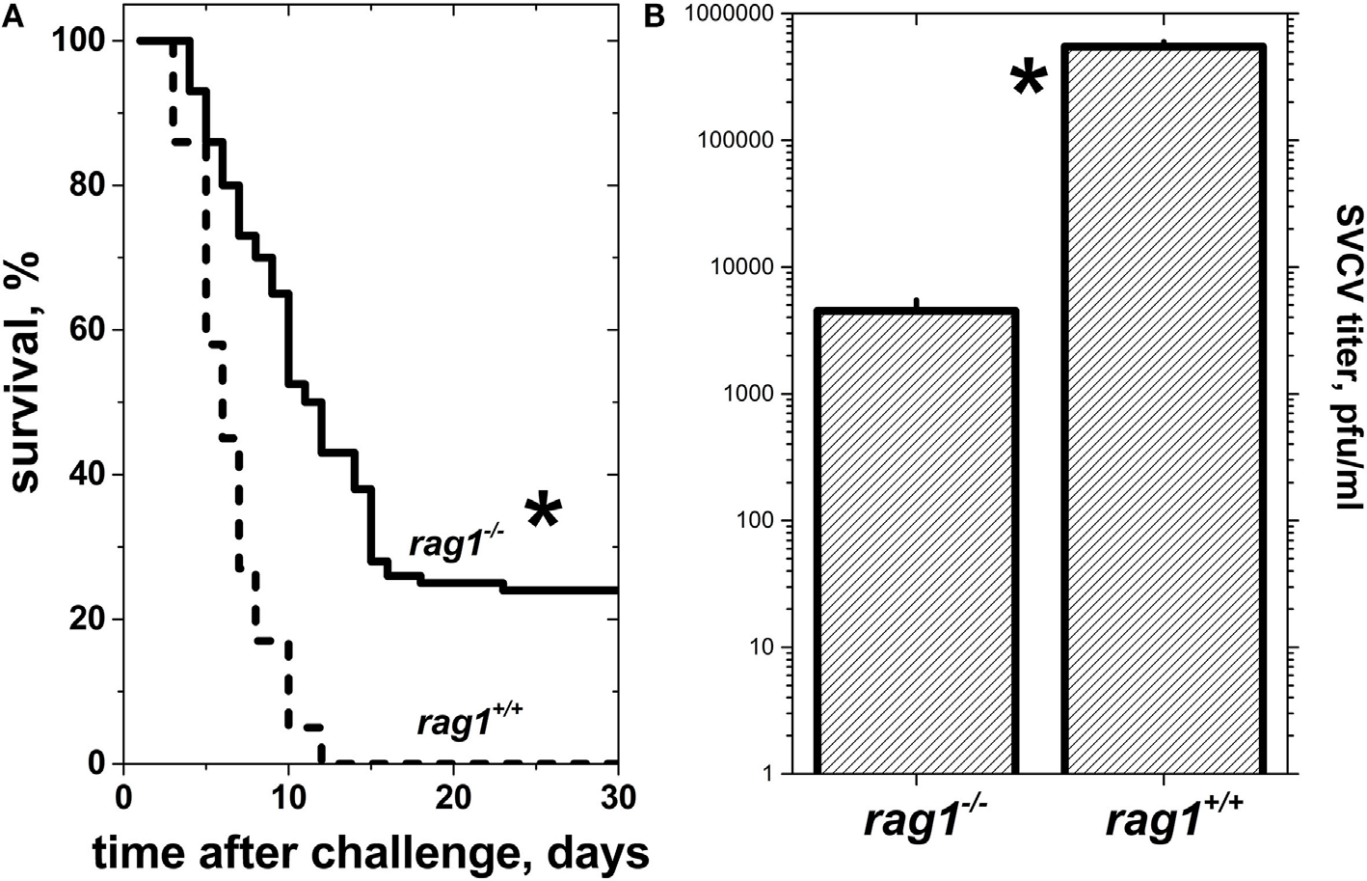
**Naïve adult *rag1*^−/−^ zebra fish are more resistant to lethal spring viremia carp virus (SVCV) infection than *rag1^+/+^* zebra fish**. **(A)** Kaplan–Meier survival curves of naïve *rag1*^−/−^ (*n* = 84) and *rag^+^*^/+^ (*n* = 100) adult zebra fish after exposure to a lethal dose of 10^4^ pfu/ml of SVCV under the same challenge conditions as in panel **(A)**. *Statistically significant differences between survival curves between *rag1*^−/−^ and *rag^+^*^/+^ as determined by the Gehan–Breslow–Wilcoxon test with *p* < 0.05 (*n* = 2). Solid lines, *rag1*^−/−^ zebra fish larvae. Dashed lines, *rag^+^*^/+^ zebra fish larvae. **(B)** SVCV titers from pooled whole zebra fish (*n* = 4 zebra fish per genotype) 3 days after SVCV infection as determined by plaque forming unit (pfu) assays.

When naïve *rag1*^−/−^ or *rag1^+/+^* zebra fish of 6 months of age were infected by bath immersion with 10^4^ pfu/ml of SVCV, there were neither infection symptoms nor deaths for the first 2 days after infection. At 3 days post-infection, zebra fish began to show external hemorrhagic symptoms in mouth, gills, lateral skin, and/or fin bases. Later on, 40–45% of adult *rag1*^−/−^ zebra fish survived infection, while 100% of *rag1^+/+^* died in the first 12 days after infection (Figure 2A). Lower viral titers (~100-fold lower) observed in *rag1*^−/−^ fish organs correlated with their delayed and lower mortality, compared to *rag1^+/+^* zebra fish (Figure 2B). Together, the mortality rates and viral loads observed in the viral challenge experiments suggest that *rag1*^−/−^ individuals were capable of mounting an enhanced innate immune response in the absence of adaptive immunity, compared to fully immunocompetent *rag1^+/+^* zebra fish. Alternatively, the absence of antibody responses in *rag1*^−/−^ could have caused less non-specific inflammation and less organ damage resulting in lower the mortality in this group. On the other hand, it is also possible that limitations of the antiviral innate response imposed by the adaptive immune system (i.e., antibodies) could explain higher mortalities in the *rag1^+/+^*.

### Increased Resistance to SVCV Infection in *rag1*^−/−^ Adult Zebra Fish Is Accompanied by Increased Transcript Expression of Innate Immune-Related Genes in Skin and Fins

**Figure 3:**
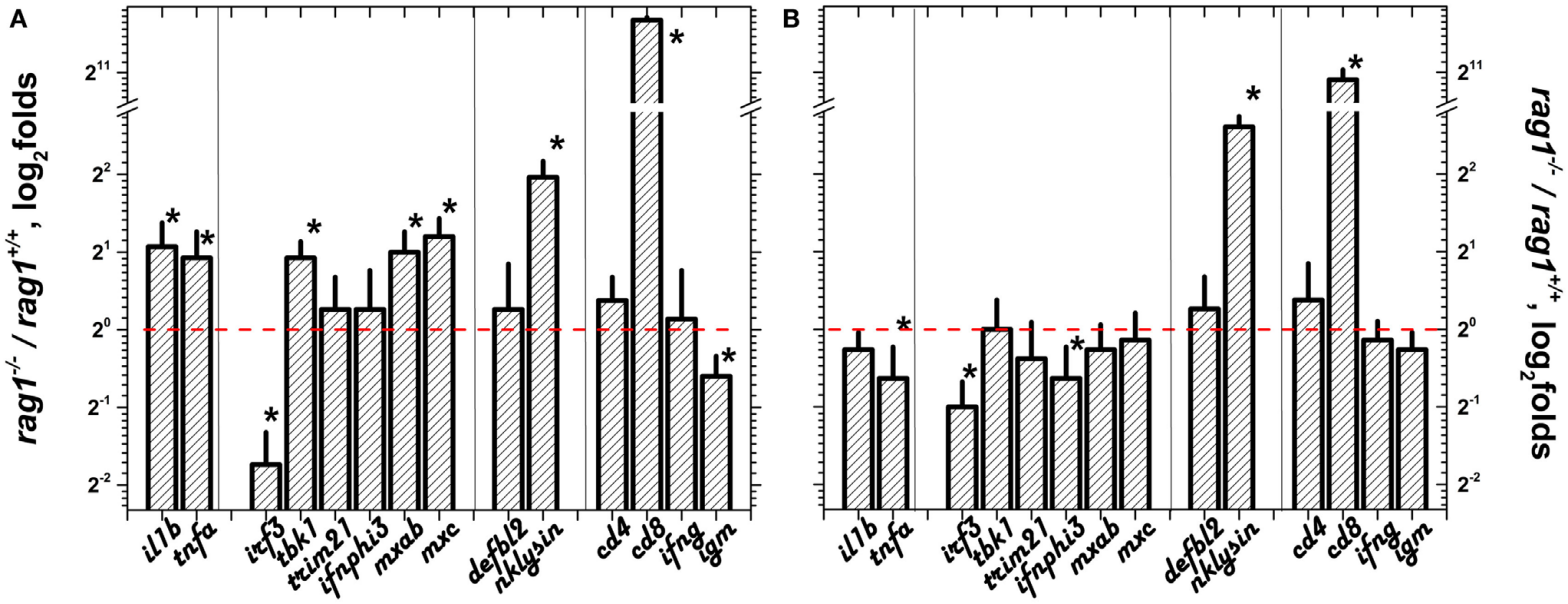
**Differential expression in skin/fins of adult zebra fish before and after spring viremia carp virus (SVCV) infection**. The transcript differential expression folds of *rag1*^−/−^/*rag1^+/+^* adult zebra fish in skin/fins were calculated as in Figure 1: **(A)** before infection and **(B)** 3 days after SVCV infection. Means and SDs were represented (*n* = 4–6 zebra fish). Red dashed line, onefold boundary. *Statistically different from onefold with *p* < 0.05 by the *t*-test.

Because previous reports suggested the importance of the skin barrier in protecting zebra fish against viral infection and skin and fins are principal entry sites for SVCV (17, 47, 51), we studied the selected transcript differential expression in these organs. Before infection, all of the genes in the sGS except *irf3* were upregulated in skin/fins of adult *rag1*^−/−^ (Figure 3A) with *nklysin* and *cd8* being the most upregulated. After SVCV infection, however, most of the individual gene differential expressions were reduced except for *irf3* and *nklysin* (Figure 3B). To note that *cd8* and *nklysin*, a marker for T cytotoxic cells and a gene highly expressed in both cytotoxic and NK-cells, respectively (79), were both upregulated before and after SVCV infection in *rag1*^−/−^ zebra fish, suggesting that the participation of these cellular types are important in the antiviral response in these fish. These results indicate that transcription of the sGS genes is more active in naïve *rag1*^−/−^ skin/fins fish than in *rag1^+/+^*, an advantage for resisting SVCV infection. It is also worth noting that transcript expression folds for the genes upregulated in adult *rag1*^−/−^ skin/fins correlated with folds of *rag1*^−/−^ larvae with a Pearson’s coefficient of 0.63 (using a polynomial fit) but not with those from embryo eggs (data not shown). Presently, whether the mechanisms that lead to the acquired overexpression of these genes in *rag1*^−/−^ larvae involve epigenetic changes or other mechanisms is being evaluated but, for now, this is beyond the scope of this study.

### Apoptosis-, Multigene Family, and Interferon-Related Genes Are Upregulated in Uninfected *rag1*^−/−^ Adult Zebra Fish Lymphoid Organs

To further investigate the transcriptomic profile behind the enhanced survival of naïve adult *rag1*^−/−^ after SVCV infection, transcript expression was estimated using both immune-targeted, in-house-designed, and commercially available genome wide microarrays. Analysis was performed in transcripts from pooled head kidneys (the analog of mammalian bone marrow) and spleens before and after SVCV infection or after poly(I:C) injection (to mimic viral replication intermediates). A total of 104 immune-related GSs and/or pathways obtained from keyword searches, KEGG, WIKI, and LEGA (see methods) were used to perform GS enrichment analysis (GSEA) and modulated multi-pathway gene (mMPG) analysis.

Table 1 shows that 25 out of the 104 GS were significantly modulated in at least one of the genotype/SVCV-infected phenotypes analyzed by GSEA using data obtained from the immune-targeted, in-house-designed microarrays. Of those 25 modulated GS, 18 (72%) were upregulated in uninfected *rag1*^−/−^ fish, including 3 GS containing genes related to apoptosis. Upregulation of apoptosis-related genes in adult *rag1^−/−^* zebra fish is described here for the first time (Table 1, gray rows, columns A and 2). In addition to apoptosis, the multigene families “NITR” and “MX” (upregulated) or “CRP” and protein degradation *psm* genes (downregulated), which were previously associated to transcriptional changes in VHSV-survivor zebra fish phenotypes before and after reinfection (26), were also modulated in *rag1*^−/−^ fish (Table 1, columns A and 4). Additionally, six upregulated GS contained well known interferon-related genes such as Toll-like receptors (*tlr*), interferons (*ifn*), and myxovirus-induced (*mx*) (Table 1, columns A and 1) or complement-related genes (Table 1, columns A and 3). Upregulation of interferon-related genes confirmed some of the data obtained in skin/fins in uninfected *rag1*^−/−^ fish (Figure 3A), strongly suggesting that a systemic upregulation of many genes traditionally implicated in the response to viral infection occurred in the absence of virus. Other GS described here for the first time in relation to uninfected *rag1*^−/−^ innate immunity were two GS related to chemokines (“CXCS” and “CHK”) (Table 1, column A).

**Table 1:** **Comparison of significant normalized enrichment scores (NESs) of gene sets (GSs) for *rag1*^−/−^ genotype/spring viremia carp virus (SVCV)-infected phenotypes of adult zebra fish by gene set enrichment analysis (GSEA)**.

GS	GSor	A	B	C	1	2	3	4
5IFN + 4MX	L	2.31**	−1.53*	1.63*	X			X
7IFN + 5MX5 + 8TLR	L	2.25**	−1.50*	1.22	X			X
2348TLR + 12IFN	L	2.20**	−1.83**	0.77	X			
9CXCS (chemokines)	L	2.13**	−1.42	0.96				
MX (myxovirus-induced proteins)	KW	2.13**	−1.56	0.95	X			X
IFN (interferon)	KW	1.93**	−1.25	1.50	X			
278CASP (caspases)	L	1.88**	−1.86**	−0.97		X		
NITR (novel immune-type receptors)	KW	1.88**	−1.88**	1.04				X
Type II interferon signaling (ifng)	W	1.84**	−1.43	−0.76	X			
MHC (major histocompatibility complex)	KW	1.83**	−0.88	−0.99				
7ONCOS (oncogenes)	L	1.76**	−2.01**	0.85				
7TLR7CASP (toll-like recpt + caspases)	L	1.74**	−1.28	2.20**		X		
Cytosolic DNA-sensing pathway	K	1.69**	−1.42	0.84				
Intestinal immune network IgA	K	1.62**	−1.40	1.34				
AMP (antimicrobial peptides)	KW	1.61**	1.18	1.74**				
Apoptosis modulation by hsp70	W	1.59**	−1.16	0.79		X		
CHK (chemokines)	KW	1.57**	−0.72	1.37				
1CREB (transcription factors)	L	1.46*	−1.93**	−1.43				
Proteasome degradation	W	1.19	1.98**	1.16				X
COM (complement)	KW	0.82	1.37**	1.23			X	
Complement and coagulation cascades	K	−1.03	1.94**	1.79**			X	
1NFKB2NFKBIAB (NF-kB related)	L	−1.60*	−1.06	−3.07**				
CRP (c-reactive proteins)	KW	−2.20**	1.60**	−0.68				X
7MAPKS (MAP kinases)	L	−2.30**	2.33**	−1.10				
1IG3MAPK (MAP kinases)	L	−2.40**	2.01**	−2.05**				

The immune-targeted in-house-designed microarray, Agilent’s ID47562 in a 8 × 15 K format deposited in GEO’s GPL17670 was used. Raw and normalized data were deposited in the GEO’s GSE54096. GSor, GS origin. K, GS retrieved from human orthologous KEGG pathways. W, GS retrieved from human orthologous WIKI pathways. KW, GS retrieved from the GenBank data by searching zebra fish (*Danio rerio*) mRNA with key-words, briefly described within parenthesis. L, novel GS proposed by leading edge gene analysis (LEGA) of the differential transcription profiles from different zebra fish genotypes/phenotypes, including those previously described (26). The numbers before the LEGA gene names indicate the number of those genes in each GS. The significantly modulated GS were ordered by their NES values in (A). **False discovery rate (FDR) *q* value < 0.05. *FDR *q* value < 0.25. (A) *rag1*^−/−^ versus *rag1^+/+^* genotypes. (B) SVCV-infected *rag1*^−/−^ phenotype versus *rag1*^−/−^ genotype. (C) SVCV-infected *rag1^+/+^* phenotype versus *rag1^+/+^* genotype. 1, GS of interferon-related TLR + IFN + MX genes. 2, GS related to apoptosis/caspases (gray rows). 3, GS related to complement. 4, GS related to multigene families modulated in VHSV survivors. 5, GS related to multigene families modulated in VHSV survivors.

We then compared the modulated GS after SVCV infection in both genotypes (*rag1*^−/−^ and *rag1^+/+^*) using genotype-matched uninfected zebra fish to calculate differential expression folds. In *rag1*^−/−^ fish, all of the GS that were upregulated in naïve fish were downregulated after SVCV infection (compare the 18 GS with upregulated NES in Table 1, column A with those in Table 1, column B). One possible explanation for such infection-induced downregulation could be that cell migration from internal lymphoid organs to peripheral tissues (i.e., viral entry tissues), reduces immune gene transcript levels in internal organs. On the contrary, GS that were downregulated in uninfected *rag1*^−/−^ became upregulated after SVCV infection. For example, “Proteosome degradation” and “CRP” (Table 1, columns B and 4), complement (Table 1, columns B and 3), and others (Table 1, columns B) indicating an infection-induced response. On the other hand, SVCV infection of *rag1^+/+^* upregulated complement (Table 1, columns C and 4), 7TLR7CASP and AMP antimicrobial peptides (Table 1, columns C) GS.

Because some SVCV proteins have immunosuppressive effects (52, 80), we decided to study the differential expression fold profiles before and after injection of poly(I:C), a model for viral double-stranded RNA short-lived replication intermediates, in the absence of viral protein and their associated immunosuppressive effects. Thirty GS were modulated in at least one of the genotype/phenotypes when tested by GSEA using genome-wide microarrays (Table 2). In *rag1*^−/−^ fish, 10 GS were upregulated, 4 of them containing genes related to apoptosis, coinciding with some of the results obtained in the same fish infected with SVCV (Table 1 gray rows, columns A and 2). In addition, 3 GS contained interferon-related genes such as *tlr, ifn*, and/or *mx* genes (Table 2, columns A and 1). Poly (I:C) exclusively upregulated the GS “EGFR1 signaling pathway”, “P13K-AKT signaling pathway” and “TGFB signaling wikipathway” (Table 2, columns A). It is worth noting that after being injected with poly(I:C), *rag1*^−/−^ fish maintained similarly modulated GS than naïve fish (Table 2, columns B, 1, and 3), as opposed to what occurred in the same fish when infected with SVCV (Table 1, columns B). Interestingly, after injection with Poly (I:C), *rag1*^−/−^ fish upregulated 5 GS participating in protein degradation pathways including, “Heat shock proteins,” “Ubiquitin mediated proteolysis,” “Protein processing in endoplasmic reticulum,”, “Proteosome degradation,” and “Protein export” (Table 2, columns B and 3). This suggests that there is a relationship between viral RNA intermediates and protein degradation pathways. In sharper contrast with SVCV infection, poly(I:C) injection of *rag1^+/+^* zebra fish, downregulated GS related to complement (Table 2, columns C and 4), suggesting opposite effects of SVCV and poly(I:C) regarding this pathway.

**Table 2:** **Comparison of significant normalized enrichment scores (NESs) of gene sets (GSs) for genotype/poly(I:C)-injected phenotypes of adult zebra fish by gene set enrichment analysis (GSEA)**.

GS	GSor	A	B	C	1	2	3	4	5
EGFR1 signaling pathway	W	1.92**	−0.68	0.62					
7IFN + 5MX5 + 8TLR	L	1.88**	2.17**	0.97	X				X
APO (Apoptosis)	KW	1.82*	1.82**	0.7		X			
Type II interferon signaling (IFNG)	W	1.77*	1.00	−1.03	X				
5IFN + 4MX	L	1.70*	2.17**	0.82	X				X
Apoptosis	K	1.69*	−0.42	0.65		X			
ApoptosisW	W	1.67*	0.78	0.67		X			
Apoptosis modulation by HSP70	W	1.65*	1.34	−0.89		X			
PI3K-AKT signaling pathway	K	1.64*	−0.63	0.55					
TGFB signaling wikipathway	W	1.64*	−1.25	−0.65					
7ONCOS (oncogenes)	L	1.59	−1.03	−0.76					
MAPK cascade	W	1.57	−0.72	0.77					
Interleukin5	W	1.56	−0.42	1.3					
BAC.MAPK + PIK	L	1.54	−1.40	−0.47					
23789CASPS	L	1.53	−0.80	0.81		X			
Alha6-beta4 integrin signaling	W	1.50	−1.62	−0.83					
278CASP	L	1.47	0.98	−1.16		X			
ONG (oncogenes)	KW	1.46	−1.02	−1.13					
FGF signaling pathway	W	1.45	−0.51	1.71					
P38 MAPK signaling pathway	W	1.44	0.74	−0.82					
MX (Myxovirus-induced proteins)	KW	1.39	1.66*	0.91	X				X
IFN (interferon)	KW	1.36	1.77*	−0.67	X				
COM (complement)	KW	1.13	−1.81*	−1.89*				X	
Complement and coagulation cascades	K	0.84	−1.15	−1.95*				X	
TLR (Toll-like receptors)	KW	0.17	1.54*	−1.18	X				
HSP (heat shock proteins)	KW	−0.76	1.55*	0.98			X		
Ubiquitin-mediated proteolysis	K	−1.12	1.66*	1.67			X		
Protein processing in endoplasmic reticulum	K	−1.14	1.56*	0.84			X		
Proteosome degradation	W	−1.43	2.55**	0.88			X		X
Protein export	K	−1.91**	2.14**	1.27			X		

The genome-wide microarray Agilent’s ID019161 in a 4 × 44 K format vs2 was used. Raw and normalized data were deposited in the GEO’s GSE91397. GSEA was performed as described in the legend of Table 1. **False discovery rate (FDR) *q* value < 0.05. *FDR *q* value < 0.25. (A) *rag1*^−/−^ versus *rag1^+/+^* genotypes. (B) Poly(I:C)-injected *rag1*^−/−^ phenotype versus *rag1*^−/−^ genotype. (C) Poly(I:C)-injected *rag1^+/+^* phenotype versus *rag1^+/+^* genotype. 1, GS of interferon-related TLR + IFN + MX genes. 2, GS related to apoptosis/caspases (gray rows). 3, GS related to proteolysis. 4, GS related to complement. 5, GS related to multigene families modulated in VHSV survivors.

Gene set enrichment analysis of the data obtained in the SVCV infection and poly(I:C) injection experiments revealed important contributions of immune-related multigene families to the rapid defense mechanisms observed in *rag1*^−/−^ fish. To some extent these results are in agreement with those from a previous study carried out with zebra fish which survived lethal VHSV infections. When reinfected, VHSV-survivor zebra fish responded more rapidly to infection due to a preceding modulation of specific innate multigene families, rather than to gene regulation in response to the virus (26). These multigene families containing 7–15 different genes each, included c-reactive proteins (*crp*), myxovirus-induced proteins (*mx*), novel immunoglobulin-type receptors (*nitr*), and proteasome subunit macropain proteins (*psm*). The regulation of these innate multigene families was indicative of responses similar to fish adaptive secondary responses, characterized by shorter lag times (1), rather than by both increased speed and magnitude of the response, as it ensues in mammal species. Because these multigene families present these similarities with adaptive memory responses and abundant gene polymorphisms (which allow for a higher variability), they are candidates for mediators of trained immunity or its equivalent in fish. Based on all the above commented results, it could be argued that in the absence of adaptive immunity, the continuous exposure to microorganisms present in the water activates compensatory innate immune mechanisms that lead to an antiviral alert state characterized by the modulation of apoptosis, multigene family and interferon-related transcripts that facilitate a more rapid and fish “adaptive”/memory-like response to pathogens, in this case a rhabdovirus.

### Identification of Modulated Genes Common to Multiple Pathways (mMPG)

To identify modulated genes with high impact on network regulation in *rag1*^−/−^ fish before and after viral infection, we performed a modulated MultiPath Gene (mMPG) analysis. In order to do this, fold data were extracted for genes that (i) were common to > 6 pathways, (ii) had a fold >1.5 or <0.66, and (iii) were significantly different (*p* < 0.05) from one of the 1.5/0.66-fold thresholds. The results showed that in uninfected *rag1*^−/−^ fish several apoptosis-related mMPG were regulated, being *fas, faslg, hsp90, casp7*, and *hspb* the most upregulated (Table 3 gray rows, column A, genes labeled with +), which confirms the importance of apoptosis for the survival of this genotype. Other important genes upregulated in the same fish included pro-inflammatory cytokine *il1b*, and the highly pleiotropic transcription factors *stat1a* and *stat3*. After infection with SVCV most of these mMPG were downregulated in *rag1*^−/−^ fish (Table 1, column B), while only *il1b, irf6*, and *hspb* were upregulated in SVCV-infected *rag1^+/+^* fish (Table 3, column C).

**Table 3:** **Modulated MultiPath Genes (mMPG) in genotype/spring viremia carp virus (SVCV)-infected phenotypes of adult zebra fish**.

Gene	#	A		B		C		Gene description	Accession numbers
Symbols		Mean	SD	Mean	SD	Mean	SD		
*hsp90*+	8	5.03*	0.79	0.20*	0.16	0.79	0.15	Heat shock protein 90. beta	NM_198210
*fas*+	9	4.09*	0.27	0.61*	0.09	1.27	0.16	Fas (TNF receptor superfamily, member 6)	XM_685355
*il1b*	14	3.71*	0.47	0.42*	0.05	2.05*	0.34	Interleukin 1, beta	NM_212844
*hspb*+	7	3.51*	0.33	0.52*	0.04	2.27*	0.32	Heat shock protein. alpha-crystalline-related	NM 001008615
*Ifih*	7	2.78*	0.47	0.48*	0.02	0.78	0.04	Novel protein similar to vertebrate interferon	XM_689032
*irf7*	8	2.65*	0.17	0.80	0.05	1.29	0.16	Interferon regulatory factor 7	NM_200677
*faslg*+	11	2.38*	0.28	0.84	0.05	0.91	0.26	Fas ligand (TNF superfamily, member 6)	NM_001042701
*casp7*+	7	2.23*	0.07	0.55*	0.04	1.36	0.11	Caspase 7, apoptosis cysteine peptidase	NM_001020607
*jak1*	17	2.21*	0.14	0.76	0.07	1.25	0.12	Janus kinase 1	NM_131073
*stat3*	13	2.17*	0.24	0.80	0.10	1.18	0.07	Signal transduction/activation transcription 3	XM_002661113
*stat1a*	23	1.93*	0.15	1.00	0.18	1.21	0.17	Signal transduction/activation transcription 1a	NM_131480
*Ikbke*	9	1.90*	0.12	0.69	0.03	1.65	0.19	Inhibitor of kappa light polypeptide gene	NM_001002751
*Tnfsf*	8	1.82*	0.54	0.73	0.21	1.16	0.39	Tumor necrosis factor (ligand) superfamily	NM_001002593
*inpp5d*	7	1.75*	0.11	0.79	0.01	1.09	0.08	Inositol polyphosphate-5-phosphatase	XM_001922972
*irf6*	8	0.73	0.12	0.90	0.29	2.15*	0.56	Interferon regulatory factor 6	NM_200598
*Igm*	7	0.03*	0.01	0.73	0.04	0.51*	0.02	Ig heavy chain constant region home design	AY643753

Modulated (differential expression folds > 1.5 or < 0.66) genes present in > 6 KEGG pathways (mMPG) were filtered/extracted from the microarray data. The mMPG were tabulated together with their corresponding mean folds (*n* = 2) and SDs, ordered by fold values in A. #, number of pathways per mMPG. The rest of the 154 MPG in the microarray were not modulated. *Significatively <0.66 or >1.5-fold at the *p* < 0.05 level by the *t*-test. (A) *rag1*^−/−^ versus *rag1^+/^*^+^ genotypes. (B) SVCV-infected *rag1*^−/−^ phenotype versus *rag1*^−/−^ genotype. (C) SVCV-infected *rag1^+/+^* phenotype versus *rag1^+/+^*genotype. + and gray rows, apoptosis-related mMPG.

As expected, the level of immunoglobulin *igm* (probes designed against its heavy chain constant domain) was downregulated in uninfected *rag1*^−/−^, compared to *rag1^+/+^* fish. However, the most intriguing data was the upregulation of *igm* in *rag1*^−/−^ fish after infection with SVCV. This might reflect a host desperate attempt to activate *igm* constant domains in the presence of a pathogen, despite the absence of recombination of the variable domains. Thus, it is also possible that although there is no V(D)J recombination in *rag1*^−/−^ mutants, some *igm* sterile transcription occurs, as previously suggested by others (81).

### Active CASPASE-3 Contributes to the Antiviral Alert State before and after SVCV Infection in Adult rag1^−/−^ Zebra Fish

**Figure 4:**
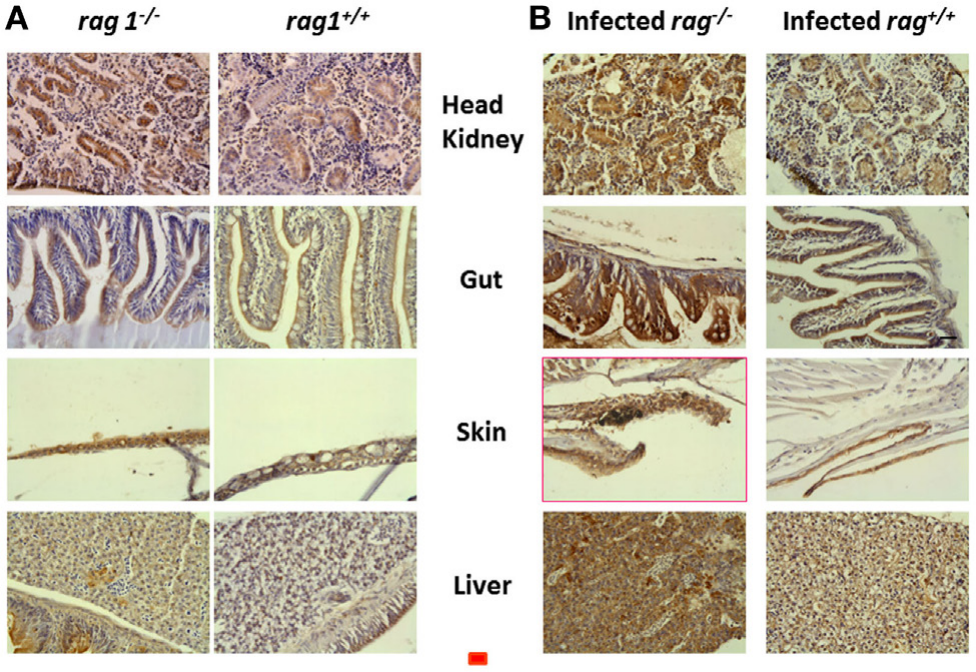
**Active CASPASE-3 staining in different tissues from uninfected and spring viremia carp virus (SVCV)-infected *rag1*^−/−^ and *rag1*^+/+^ adult zebra fish**. Tissue sections from **(A)** uninfected and **(B)** SVCV-infected *rag1*^−/−^ and *rag1^+/+^* adult zebra fish were stained by anti-human active CASPASE-3 antibody. Dark-stained areas in all *rag1*^−/−^ tissues indicate the presence of active CASPASE-3. Images are representative of at least two independent experiments. Horizontal red line, 30 μm.

Because apoptosis was likely to be important for the survival of *rag1*^−/−^ fish based on the transcript upregulations of apoptosis-related GS (Tables 1 and 2) and mMPG (Table 3), we tested whether apoptosis was activated also at the protein level. To that end, we stained zebra fish head kidney, gut, skin, and liver with an anti-apoptosis protein antibody. For those experiments, we chose active CASPASE-3 because of its amplifying role in CASPASE-8-mediated mammalian/fish apoptosis (82, 83). Staining of uninfected histological sections revealed augmented positive staining in *rag1*^−/−^ tissues compared to *rag1^+/+^* (Figure 4A). When tissues from SVCV-infected zebra fish were stained, active CASPASE-3 positive staining increased in both *rag1*^−/−^ and *rag1^+/+^* fish (Figure 4B). Therefore, it could be argued that as part of the *rag1*^−/−^ antiviral alert state both transcripts and proteins facilitate the rapid induction of apoptosis, which would help to eliminate virus-infected cells and increase survival.

### Both the Level of Immune Cell-Specific Transcripts and Leukocyte Infiltration Are Increased in Uninfected *rag1*^−/−^ Fish Tissues

Because of the lack of molecular markers for zebra fish immune cells, cGS including immune cell type-representative genes were designed as alternative markers in a previous study. The same analysis was applied here to evaluate the relative importance of each particular cell type in the *rag1*^−/−^ genotype before and after SVCV infection.

Transcripts from NK-cells and macrophages were the most upregulated cGS in uninfected *rag1*^−/−^ fish (Table 4). Both NK-cells and macrophages have been identified in higher vertebrate models, as mediators of trained immunity (9, 11, 16, 18). In partial agreement with our results, *rag1*^−/−^ zebra fish developed immune memory when immunized to *Edwardsiella ictaluri* (a pathogenic bacterium) through the participation of NK-cells (25). NK-cells are traditionally defined as cells of the innate immune system because they lack RAG recombinase-dependent antigen receptors. The comparison of candidate mechanisms for mediating mammalian antigen-specific NK cell memory with other examples of RAG-independent pathways that generate antigen receptor diversity in non-mammalian species such as zebra fish, suggests that specific subsets of NK cells can develop long-lived and specific memory to a variety of antigens independent of B cells and T cells (13, 84). Most recently, antigen-specific NK cell responses to influenza and HIV viruses were induced in primates after infection and/or vaccination, an important opening for human vaccine improvement (13–15). T helper cell 17 (TH17) and TH2 were also upregulated in uninfected *rag1*^−/−^ fish, contrary to other cellular types such as B-cells and neutrophils. Interestingly, all head kidney/spleen cellular types studied were downregulated in *rag1*^−/−^ fish after SVCV infection, suggesting again that cellular migration to peripheral tissues ensues after infection. In contrast, in *rag1^+/+^* fish, B-cells and neutrophils were upregulated (Table 4).

**Table 4:** **Comparison of normalized enrichment scores (NESs) obtained by using gene set enrichment analysis (GSEA) of cellular gene sets (cGS)**.

cGS	No. of genes per cGS	A	B	C
NK-cells	35	1.83**	−1.61**	−1.03
Macrophages	31	1.60*	−1.48*	0.96
TH17	37	1.51*	−1.58**	1.19
TH2	31	1.47*	−1.51*	1.11
Dendritic	10	1.29	−1.28*	1.05
BZ-cells	23	1.25	−1.08	1.51
B-cells	23	1.18	−1.03	1.47*
TH1	30	1.17	−1.64*	−0.93
Neutrophil	16	1.11	−0.86	1.44*
Treg	25	0.96	−1.44*	−1.12
CTL	12	−0.84	−1.04	−1.40

The table shows the NES of each cGS on the different zebra fish genotype/spring viremia carp virus (SVCV)-infected phenotypes ordered by those of A. NK-cells, natural killer cells; macrophages, monocyte/macrophages; Th17, T-helper 17 cells; Th2, T-helper 2 cells; dendritic, dendritic cells; BZ cells, mucosal IgZ producing cells; B cells, serum IgM producing cells; Th1, T-helper 1 cells; Neutrophil, neutrophil and granulocyte cells; Treg, T regulatory cells; CTL, antigen-specific cytotoxic cells. **False discovery rate (FDR) q value < 0.05. *FDR q value < 0.25. (A) rag1^−/−^ versus rag1^+/+^ genotypes. (B) SVCV-infected rag1^−/−^ phenotype versus rag1^−/−^ genotype. (C) SVCV-infected rag1^+/+^ phenotype versus rag1^+/+^ genotype. Note that B-cells were upregulated only in SVCV-infected rag1^+/+^.

**Figure 5:**
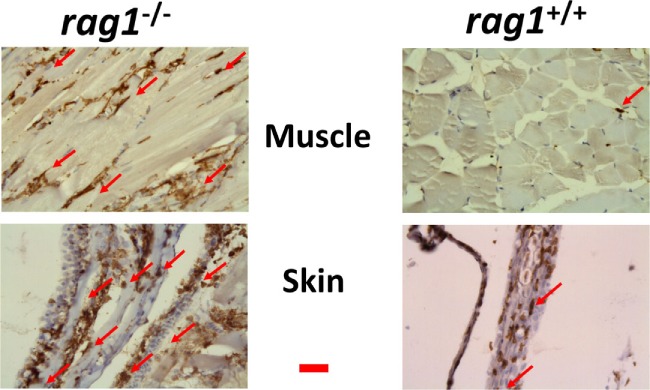
**Leukocyte infiltration in muscle and skin tissues in *rag1*^−/−^ and *rag1^+/+^* adult zebra fish**. Histological sections from muscle and skin tissues of *rag1*^−/−^ and *rag1^+/+^* were stained with an antibody anti-l-plastin, a pan-leukocyte marker (dark-stained areas). Red arrows, examples of l-plastin stained cells. Images are representative of at least two independent experiments. Horizontal red line, 30 μm.

Since the transcript data suggested that NK-cells and/or macrophages might infiltrate *rag1*^−/−^ tissues, we stained the corresponding zebra fish tissue sections with an anti-l-plastin polyclonal antibody, a pan-leukocyte marker (85, 86), to explore for the presence of leukocytes in peripheral tissues. Leukocytes were abundant in significant amounts in zebra fish muscle and skin from uninfected *rag1*^−/−^ fish (Figure 5). In contrast, equivalent tissue sections from *rag1^+/+^* fish showed limited leukocyte presence (Figure 5), confirming previous reports (45).

After recognizing different pathogen-associated molecular patterns, mammalian NK-cells and macrophages undergo epigenetic changes to become effector cells for trained immunity (9, 18). Our results showed that cell-specific gene expression and immunohistology data from *rag1*^−/−^ zebra fish confirmed the increased function and/or activation of NK-cells/macrophages and leukocytes, respectively. Therefore, it could be argued that in *rag1*^−/−^ zebra fish these cellular types mediate a shorter lag time responses that resemble adaptive responses in fish, consistent with trained immunity responses mediated by similar cells in other vertebrate models.

## Discussion

In this work, we show that the *rag1*^−/−^ genotype that results in deficient adaptive immunity in zebra fish, favored the development of an acquired antiviral alert state that correlated with increased resistance to lethal infection with SVCV. A plausible explanation for the pre-existing antiviral alert state observed in uninfected *rag1*^−/−^ fish could be that the continuous exposure to aquatic microbiota (present in the aquaria) induces increased and perhaps species-specific variation in transcriptional levels. Similarly, a continuous exposure to the remaining, latent virus could be the cause for the maintenance of similar antiviral states in immunocompetent zebra fish that survived VHSV infection (a related fish rhabdovirus infection) (26). Alternatively, the lower viral loads in *rag1*^−/−^ fish could be reflecting that in the absence of adaptive immunity and antibodies, the innate immune response develops unchecked. On the contrary, in immunocompetent *rag1^+/+^* zebra fish, adaptive mechanisms (e.g., antibodies) would limit the innate antiviral response. In this hypothesis, the *rag1^+/+^* downregulation of innate genes may occur as a feed-back mechanism to prevent the host cell damage otherwise induced by uncontrolled upregulation of the innate response. If such feedback mechanisms are mediated by IgM antibodies, they would involve immunoglobulin receptors, hypothesis which could be tested by injecting zebra fish IgM into *rag1*^−/−^ fish. In this context, it is known that in mammalians secreted IgG levels sensed by B-cell receptors (FcγRIIB) regulate IgM plasma levels (87). Since in zebra fish (88) or in any other fish (89, 90), only polymeric Ig receptors (PIGR/*pigr*) have been identified, any IgM feedback fish mechanism to control innate response levels through antibodies would, in principle require PIGR receptors. Therefore, anti-PIGR antibodies could also be used to block an hypothetical IgM feedback. Future experimentation addressing this and/or other hypothesis will help clarify the mechanism for maintaining the acquired adult *rag1*^−/−^ fish antiviral alert state.

This acquired *rag1*^−/−^ antiviral alert state included modulation of previously described immune multigene families and interferon-related genes in VHSV-survivor zebra fish, but the participation of apoptosis and putative NK-cells/macrophages (some of the characteristics of mammalian trained immunity) is described here for the first time. Nevertheless, although we introduced an original way to detect putative NK cells by using their transcript expression profiles (26), at present we cannot validate those results, since there are no zebra fish specific reagents available. On the other hand, resemblance in the modulation of multigene families (*mx, crp, nitr, psm*) between *rag1*^−/−^ fish and VHSV-survivor *rag1^+/+^* zebra fish, suggests that those genes are important contributors to similar acquired antiviral alert states in both cases (genotypic and phenotypic, respectively). Because, some of the multigene families modulated in *rag1*^−/−^ and survivor rag1^+/+^ zebra fish (i.e., *nitr*) have orthologs linked to NK-cell memory in mammalian trained immunity (35), they might also be candidates for mediators of trained immunity or its equivalent in fish species.

As mentioned above, we have described apoptosis in *rag1*^−/−^ fish as a factor that may contribute to the maintenance of their acquired antiviral alert state. The inhibition of early immune response of fish hosts by species-specific non-virion novirhabdoviral proteins (80, 91) or by other viral proteins in fish herpes virus CyHV-3 and/or SVCV (92), underline the importance that apoptosis or other alternative immune pathways may have as rapid mechanisms of defense against viruses in fish. The presence of high levels of apoptosis-related transcripts and activated CASP-3 protein strongly suggests that apoptosis is associated with enhanced viral protection of *rag1*^−/−^ zebra fish, possibly by eliminating viral-infected cells at an early stage of infection. In this regard, recent system biology approaches have confirmed that similar early pathogen/zebra fish crosstalks may explain the final outcome of many infections (93). In contrast, late apoptosis-dependent lysis of host cells favors viral spread, as described for several fish rhabdoviruses, including SVCV (92, 94–96), which indicates that a prompt activation is crucial for apoptosis to exert its antiviral role during rhabdoviral infections.

In this work, we have gathered immunohistochemical evidence that demonstrates that *rag1*^−/−^ zebra fish maintain elevated numbers of leukocytes in peripheral tissues, compared to *rag1^+/+^* zebra fish. The differences are maintained before and after SVCV infection correlating with the increased resistance of *rag1*^−/−^ fish to infection and the transcriptional downregulation of some immune genes in internal, lymphoid organs. All these results could be indicative of zebra fish cell migration (from lymphoid organs) as one of the mechanisms for eliciting rapid responses to viral infection. Thus, similar leukocyte cell migration has been previously shown to be altered in zebra fish during disease (97) and B-cell migration explained the high levels of plasma neutralizing antibodies in VHSV-survivor zebra fish coinciding with B-cell (IgM + cells by flow cytometry) depletion in lymphoid organs (26).

A growing number of studies in mammalian models have identified vaccine non-specific side effects, which may be explained by trained immunity. Non-specific effects of viral (e.g., measles or oral polio) and bacterial (BCG vaccine against tuberculosis or the diphtheria-tetanus-pertussis) vaccines have been shown to affect the survival of children with different outcomes (i.e., increased or decreased mortality) depending on the vaccine (98). Most recently, human studies showed that BCG immunization primes the immune system of adult individuals so that the subsequent BCG-unrelated immune responses to an influenza vaccine are enhanced, a phenomenon attributed to trained immunity (99). These studies and others suggest that non-specific vaccination effects could be harnessed to improve overall health. Our work shows how adult, adaptive immunity-deficient zebra fish exhibit increased resistance to a lethal viral infection with SVCV, contrary to larvae and most importantly adult, immunocompetent zebra fish. However, regardless of what event(s) or cue(s) trigger the development of enhanced immunity to SVCV in *rag1*^−/−^ zebra fish, this model, integrating *rag1*^−/−^ zebra fish and subsequent infections reproduces, at least to some extent, the development of non-specific, protective immunity *in vivo*. However, challenges with heterologous pathogens should be performed in the future to obtain evidence for such protectio against non-specific pathogens.

On the other hand, trained immunity in mammalians is associated to epigenetic reprogramming (e.g., cytosine methylations, histone acetylation/hypermethylation, or miRNA) (10, 12, 100) rather than genetic recombination of adaptive immune receptors (20). Here, we described permanent changes in the expression of immune genes in *rag1*^−/−^ zebra fish, which resemble gene expression profile changes in zebra fish that survive lethal viral infections. Epigenetic changes in the vicinity of the promoters of these genes could explain, at least partially, how the baseline transcript expression is changed permanently in response to environmental stimuli (e.g., virus infection or microorganisms present in the water), as these genomic regions would be more or less accessible to transcription factors resulting in up or downregulation of specific genes. However, our knowledge of fish trained immunity (or its equivalent) and the availability of reagents and methods to investigate epigenetics in fish models remain scarce. For instance, in mammals, both the adaptive and trained immunity secondary responses (memory) are characterized by increased speed and magnitude, compared to primary responses (10, 12, 100). In contrast, fish adaptive secondary responses have been defined as more rapid but not bigger in magnitude than primary responses (1). Therefore, it would not be surprising that the observed increased survival of uninfected *rag1*^−/−^ zebra fish, which clearly exhibit an antiviral alert state, was a product of the promptness and not magnitude of the response to SVCV. Overall, our results strongly indicate that maintaining elevated levels of innate immune transcripts/proteins, including apoptosis effector molecules, may be an efficient mechanism to provide rapid protection against lethal virus infections in the aquatic environment, at least in the absence of adaptive immunity (this work) and in fish, which have survived virus infections (26). The possible epigenetics that may be implicated remains to be investigated. The results of the studies reported here, in turn, raise unexpected questions, such as, is there a common on/off epigenetic switch triggering a unique antiviral alert state in both uninfected *rag1*^−/−^ and viral survivor fish? Or, is the premature physical deterioration/aging observed in adult *rag1*^−/−^ fish (Figure S1 in Supplementary Material) a physiological cost for maintaining a permanent antiviral alert state? In the future, studies such as this and similar future experimental setups using *rag1*^−/−^ zebra fish should help answer these questions and other in relation to innate immunity in fish species.

## Author Contributions

PG-V performed infection experiments, RTqPCR at Elche and help in writing the MS. AM-L performed infection experiments and RTqPCR at Elche and wrote the first drafts. AL-M performed larval infection and immunohistochemical experiments at Murcia. MB-P performed infection experiments and care of rag mutants at Elche. RM-G performed infection experiments and care of rag mutants at Elche. MO-V controlled infection experimental set up at Elche. MV performed injection experiments and care of rag mutants at Vigo. AF analyzed the microarray data at Vigo. VM coordinated the larval infection and immunohistochemical experiments at Murcia and contributed to the writing of the MS. BN coordinated rag production at Vigo and contributed to the writing of the ms. AE coordinated the whole work at Elche and contributed to the writing of the MS. JC performed and analyzed microarray data at Madrid and coordinated and wrote the final MS.

## Conflict of Interest Statement

The authors declare that the research was conducted in the absence of any commercial or financial relationships that could be construed as a potential conflict of interest.
