# Corrigendum: Zebra Fish Lacking Adaptive Immunity Acquire an Antiviral Alert State Characterized by Upregulated Gene Expression of Apoptosis, Multigene Families, and Interferon-Related Genes

**DOI:** 10.3389/fimmu.2017.00668

**Published:** 2017-06-13

**Authors:** Pablo García-Valtanen, Alicia Martínez-López, Azucena López-Muñoz, Melissa Bello-Perez, Regla M. Medina-Gali, María del Mar Ortega-Villaizán, Monica Varela, Antonio Figueras, Víctoriano Mulero, Beatriz Novoa, Amparo Estepa, Julio Coll

**Affiliations:** ^1^Departamento de Bioquímica, Universidad Miguel Hernández de Elche (UMH), Alicante, Spain; ^2^Facultad de Biología, Departamento de Biología Celular e Histología, Universidad de Murcia, IMIB-Arrixaca, Murcia, Spain; ^3^Instituto deInvestigaciones Marinas (IIM), Consejo Superior de Investigaciones Científicas (CSIC), Vigo, Spain; ^4^Departamento de Biotecnología, Instituto Nacional Investigación y Tecnología Agraria y Alimentaria (INIA), Madrid, Spain

**Keywords:** zebra fish rag1^−/−^ adaptive deficient mutants, spring viremia carp viral infections, multigene families and apoptosis in resistance to viral infections, trained immunity NK/macrophages in fish, antiviral alert state

In the original article there were two inaccurate statements, in the introduction and method sections, which may lead to misunderstanding the way previous microarray profiles of uninfected rag1^−/−^ zebrafish were studied by Jima et al. 2009 (ref 49 in the paper). The meaning should be clear that in previous work, rag1^−/−^ zebrafish were compared to heterozygous rag^+/−^ while in the present work they were compared to homozygous rag1^+/+^. The authors sincerely apologize for those inaccurate statements. These errors did not change the scientific conclusions of the article in any way.

The correct version of the introduction 3rd paragraph, statement on line 25 should more accurately read:

Probably, due to the difficulties encountered when breeding rag1^−/−^ zebra fish, their gene expression profiles in response to viral infection, has yet to be compared to rag1^+/+^.

The correct version of the materials and methods, zebra fish (*Danio rerio*) section, statement on line 13 should now be more accurate as:

These difficulties may explain why few people could make experiments with them and why only comparisons of rag1^−/−^ to heterozygous rag1^+/−^ have been used for microarray analysis (49).

## Conflict of Interest Statement

The authors declare that the research was conducted in the absence of any commercial or financial relationships that could be construed as a potential conflict of interest.

