# Supplementary figures and images for: Zebra Fish Lacking Adaptive Immunity Acquire an Antiviral Alert State Characterized by Upregulated Gene Expression of Apoptosis, Multigene Families, and Interferon-Related Genes
Source: Front Immunol. 2017 Feb 13;8:121. doi: 10.3389/fimmu.2017.00121 (PMC5303895; doi:10.3389/fimmu.2017.00121)

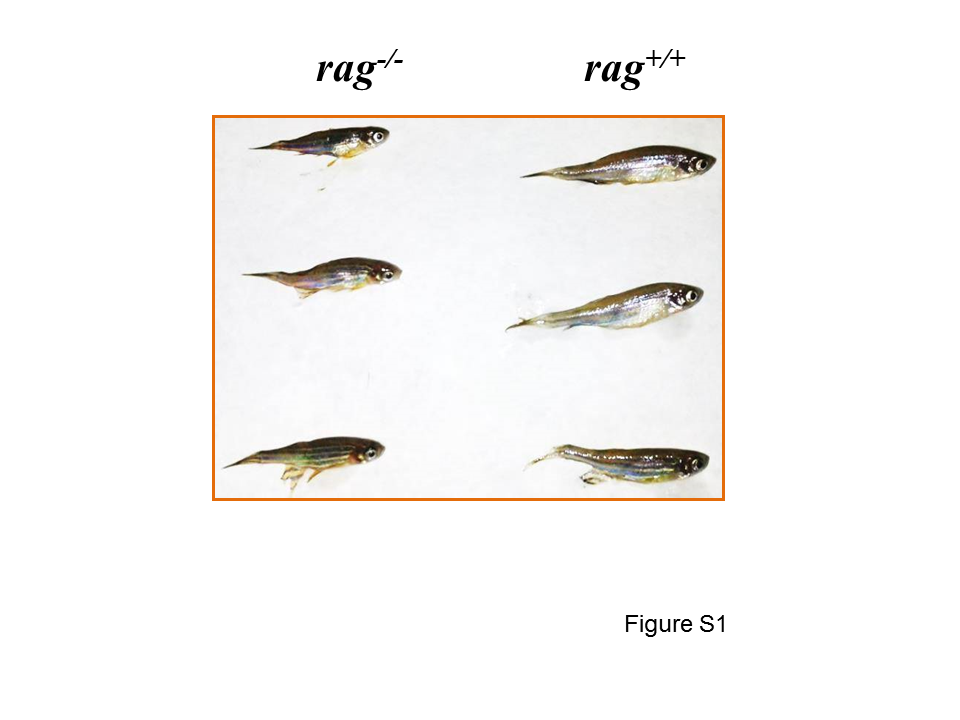

Supplement: Supplementary file 1 [file Image_1.TIF]
