# Supplementary material for: Zebra Fish Lacking Adaptive Immunity Acquire an Antiviral Alert State Characterized by Upregulated Gene Expression of Apoptosis, Multigene Families, and Interferon-Related Genes
Source: Front Immunol. 2017 Feb 13;8:121. doi: 10.3389/fimmu.2017.00121 (PMC5303895; doi:10.3389/fimmu.2017.00121)
Supplement: Supplementary file 2 [file Table_1.PDF]

Table S1

Primer sequences used for RTqPCR of sGS

**Gene**	**Forward primer (5′ → 3′)**	**Reverse primer (5′ → 3′)**	**GeneBank Accession Number**
il1b	TCATCATCGCCCTGAACAGA	CATGTCCAGCACCTCTTTTTCTC	NM 212844
tnfa	AAGCCACTTTTCAGTGCAATCC	AGCGCCGAGGTAAATAGTGTTG	NM 212859
irf3	CCCTGGAAACACGCTTTGA	GAGCCCACGCCTTGAATATG	NM_001143904
tbk1	GGGAGTTTGAGGTGCTGAAGAA	CTCGACGGCGAACAGCTT	NM_001044748
trim21	ACGCACACCTCCACAATTCA	GCTGTACGATTGAGACACTCATCTAAT	XM_003198527.3
ifnphi3	GACTGCGGGAGGGCTTTT	TTTTGCGGGAATGGTATAGAAAC	NM_001111083
mxab	GGTCTCTGGGAGTCGAAAAGG	AACTCTTTCCCGAGCTTTGGT	NM 182942-AJ544824
mxc	AAGAGCCCTGCCTAAGGTTGT	ATTTCACACCCAGACGGAACTC	NM_001007284.2
defbl2	AATGTGCATAATGCCGAAGTACA	ACAACCATGGTGAGCAACAATATATT	NM_001081554.1
nklysin	CAATGGGAGATGCACAAAGAAC	CACATCCCAGGCAGTTGCT	NM 212741
cd4	GAGTGGTGGTCTTCATCTTGCTT	GTCGTCTGTACATCATCATCTGTTTG	NM_001135096.1
cd8	CGCAAAGCAGACGGAAGTC	TGTCGATGTGCCCACTATATTTTT	BC162235.1
ifng	TTGCCAGGCTGTGTTGCTT	TTTATTATCTGACTTGTTCATCATGTCTTG	AB194272.1
igm	TCGGTTTGTCTCAGTGCAGTTC	TCTGCAGGTGAGAAACCTTTAGC	BC154613.1
ef1a	CCACGTCGACTCCGGAAA	CGATTCCACCGCATTTGTAGA	NM 131263

The *ef1a* gene was used for normalization purposes
